# Atlanta Medical College

**Published:** 1876-03

**Authors:** 


Atlanta Medical College.—The Commencement exercises of the Atlanta Medical College came off at DeGive's Opera House, on the evening of the 1st of March, and, from the Dean's report, we infer that the past session has been entirely satisfactory to the Board of Trustees and Faculty. In addition to the large class in attendance on the regular course of medical instruction, the Pharmaceutical Department established two years ago is becoming a prominent feature of this institution. The large number in attendance the present session on the pharmaceutical course, is the greatest evidence of the neceesity of such instruction in the South. In the two departments there were forty-eight graduates, a marked increase on the graduating class of the past years.

